# Mobile health education for salt reduction and hypertension prevention: effects on knowledge acquisition and behavioral change among Chinese third-grade students

**DOI:** 10.3389/fpubh.2025.1600342

**Published:** 2025-10-22

**Authors:** Yang Zhang, Jiajin Lin, Chen Qu, Tao Mao, Jiao Zhong, Xin Yuan, Yan Liu, Shiqi Zhen

**Affiliations:** ^1^Zhenjiang Center for Disease Control and Prevention, Zhenjiang, Jiangsu, China; ^2^Jiangsu Provincial Center for Disease Control and Prevention, Nanjing, Jiangsu, China

**Keywords:** mobile health education, salt reduction, hypertension prevention, knowledge acquisition, behavioral change

## Abstract

**Background and aims:**

This study evaluates the impact of a comprehensive health education intervention delivered via a mobile application on health knowledge and behavior among third-grade students in Zhenjiang, Jiangsu Province, China. With increasing rates of non-communicable diseases, particularly hypertension among children, this intervention aimed to enhance health literacy and promote healthier behaviors.

**Methods:**

A total of 15,656 students from 88 primary schools participated, with 11,614 completing both pre- and post-intervention surveys. The intervention included 20 animated health education lessons covering topics such as balanced diets, personal hygiene, and salt reduction, alongside activities involving parents and school staff.

**Results:**

Results indicated significant improvements in health knowledge, with an average increase of 5.6 points, and health behavior scores increased by 2.6 points post-intervention. The awareness of key knowledge areas, including vision protection and correct coughing/sneezing habits, had increased by more than 10%. However, improvements in health behaviors were less pronounced, underscoring the challenge of translating knowledge into action. A mixed-effects model analysis revealed that baseline scores significantly influenced post-intervention outcomes, with urban students outperforming their rural counterparts.

**Conclusion:**

This study highlights the effectiveness of mobile health education interventions in enhancing health literacy among children and suggests the need for continued focus on behavior change strategies to complement knowledge acquisition.

## 1 Introduction

Globally, non-communicable diseases (NCDs) account for approximately 71% of global annual mortality, equating to 41 million deaths (1). Among these, cardiovascular diseases (CVDs) are particularly prevalent in low- and middle-income countries (2). Hypertension serves as a significant predictor of both the incidence and mortality associated with CVDs (3), contributing substantially to the cardiovascular disease burden in China (4) where it was responsible for nearly 40% of deaths in 2017 (5). The prevalence of hypertension among adults in China is estimated at 27.9% (6), a condition exacerbated by excessive dietary salt consumption. Concurrently, the national prevalence rate of hypertension among Chinese children is as high as 14.5% and is rising, with notably higher rates observed in boys and older children (7) Given that childhood hypertension often persists into adulthood (8), early intervention is imperative to mitigate long-term health consequences (9).

Global burden of disease analyses indicate a consistent increase in the disease burden attributable to individual behaviors and lifestyle choices in China, with an imbalanced diet identified as primary contributor to morbidity and mortality. Among dietary factors, excessive sodium consumption emerges as a critical behavioral determinant (10). Empirical evidence underscores that dietary salt intake is a key determinant of elevated blood pressure, which tends to rise with advancing age and is strongly correlated with salt consumption (11, 12). Research further suggests that a preference for salty flavors is a learned behavior, with dietary patterns established during childhood persisting into adulthood (13). Consequently, initiating salt reduction interventions early in childhood, prior to the development of a salt preference, yields more substantial long-term health benefits (14, 15). A substantial body of literature supports the efficacy of integrated health promotion programs that engage both educational institutions and families in enhancing health knowledge and behaviors among students and their caregivers. For example, Cotter et al. (16) implemented a six-month educational intervention targeting salt reduction among children aged 10 to 12 in northern Portugal. The findings revealed that while all intervention groups exhibited reductions in daily salt intake, only cohort receiving a combined theoretical and practical approach demonstrated a statistically significant decrease (17).

School-based interventions have yielded substantial health benefits across various cultural settings. In the United States, a virtual cooking program aimed at low-income families in Missouri demonstrated that nutrition education facilitated through schools, when integrated with practical home cooking skills, significantly increased vegetable consumption among diverse ethnic groups, with increases of 37% among Latinx participants and 29% among African American participants. This finding underscores the cross-cultural efficacy of such interventions through active parental involvement (18). Similarly, in Australia, the Time for Healthy Habits trial evaluated translated, parent-focused programs delivered online versus in print for families with children aged 2 to 6 years. The trial reported an 89% rate of parental engagement and a 15% enhancement in parental health knowledge, highlighting the effectiveness of preference-based delivery methods in promoting healthy eating and physical activity (19).

Comparable successes have been observed in salt reduction initiatives: The pioneering New Zealand-led School-EduSalt trial (also known as the Changzhi project), conducted over 3.5 months across 28 schools in China, formally reduced salt intake and blood pressure, The New Zealand-led School-EduSalt trial in China achieved reductions in salt intake and blood pressure, thereby providing proof of concept for knowledge transfer models within this context (20). Expanding upon this global framework, the George Institute initiated targeted interventions beginning with the School-EduSalt trial (21). This “small hands leading big hands” approach resulted in a 25% reduction in salt consumption and corresponding decreases in blood pressure by employing teacher-led instruction coupled with child-to-family knowledge transfer. However, the model encountered challenges related to scalability, primarily due to the necessity for specialized teacher involvement. To overcome these limitations, subsequent innovations were developed. Notably, the AppSalt study (2017) incorporated mobile technology, achieving over 90% lesson completion rates and demonstrating a downward trend in salt intake through the use of parent-oriented applications and interactive features. These findings substantiate the feasibility of technology-mediated intervention delivery.

Ongoing challenges, including teacher shortages and the fragmentation of curricula across various initiatives, underscore the necessity for sustainable interventions. In response, the China Health Education Center, the George Institute, and Queen Mary University collaboratively designed an integrated curriculum that consolidates previous evidence-based approaches: (1) the AppSalt mobile platform, which circumvents the requirement for extensive teacher training; (2) Changzhi’s child-to-family transmission model, which fosters enhanced engagement; and (3) a continued emphasis on hypertension prevention while incorporating a broader range of health competencies. This hybrid online-offline program strategically embeds salt reduction education within existing school infrastructures to facilitate scalability and directly address previously identified implementation challenges.

In 2019, the China Health Education Center, the George Institute for Global Health at Peking University, and Queen Mary University of London collaboratively initiated the EduSaltS program to establish a viable, sustainable, and scalable model for school-based salt reduction. As a pivotal component of this initiative, a study conducted in Zhenjiang, Jiangsu Province, assesses the effectiveness of salt reduction educational interventions targeted at students. The findings from this research aim to inform the development of salt reduction education strategies tailored for student populations. Moreover, the outcomes and insights derived will support the nationwide implementation and dissemination of effective practices aligned with “Healthy China Action” framework. Additionally, this evidence will serve as a valuable reference for salt reduction efforts in other regions and contribute to the broader prevention of other chronic non communicable diseases.

The “Healthy Salt Classroom” mHealth intervention evaluated in this study was developed within the comprehensive EduSaltS program framework. Its design incorporates robust evidence-based elements, seamlessly integrating the validated “small hands leading big hands” child-to-family knowledge transfer model from the successful School-EduSalt trial (20, 21) with the sophisticated mobile technology delivery platform established in the AppSalt study (21), The intervention adheres rigorously to both Chinese Dietary Guidelines and World Health Organization recommendations regarding sodium consumption. Methodologically, the program employs a strong theoretical foundation, drawing deliberately on Social Cognitive Theory (SCT) and the COM-B (Capability, Opportunity, Motivation-Behavior) framework to structure its multifaceted components. The carefully crafted animated educational content and interactive assessments were specifically designed to enhance students’ Capability (knowledge acquisition) and Motivation (self-efficacy development and outcome expectations). Complementary point-based incentive systems and structured family activities were strategically implemented to reinforce Motivation while simultaneously creating Opportunity for practical behavioral application. This theoretical architecture was deliberately selected to address the well-documented intention-behavior gap and facilitate the critical translation of knowledge into sustained behavioral change—a persistent challenge we examine comprehensively in the Discussion section.

While previous investigations, including the School-EduSalt and AppSalt trials, have established the efficacy of child-to-family knowledge transfer mechanisms and mobile health technologies in achieving salt reduction, their scalability and seamless integration within established educational infrastructures have remained formidable challenges. The present investigation addresses a critical knowledge gap by systematically evaluating a large-scale, government-implemented health education intervention that synergistically integrates these evidence-based methodologies within a real-world, city-wide administrative framework. In contrast to prior research that predominantly concentrated on isolated salt reduction outcomes, our ‘Healthy Salt Classroom’ program strategically embeds salt reduction within a comprehensive health literacy curriculum, thereby targeting a broader spectrum of health-promoting behaviors. Furthermore, through the explicit application of the COM-B (Capability, Opportunity, Motivation-Behavior) model and Social Cognitive Theory, this investigation seeks not merely to enhance knowledge acquisition but to elucidate the complex mechanistic pathways governing the translation of knowledge into sustained behavioral modification among pediatric populations—a domain requiring more rigorous empirical exploration.

## 2 Methods

### 2.1 Study population

This study was designed as a pre-post design. Due to the city-wide scale of the administrative rollout, a concurrent control group was not feasible. The evaluation therefore relies on a pre-post comparison within the intervention group. A total of 15,656 third-grade students (aged 8–10 years) were enrolled from 88 public primary schools across seven districts of Zhenjiang City, Jiangsu Province, China. To ensure representativeness, schools were stratified according to their urban or rural status. To ensure representativeness. The recruitment process was conducted through a hierarchical administrative framework: the Zhenjiang Education Bureau issued policy directives to the schools, after which school principals and head teachers organized parent-teacher meetings to distribute registration codes for the applet and to provide detailed explanations of the study procedures. The measurement instruments employed demonstrated satisfactory reliability. Specifically, the health knowledge questionnaire, comprising 20 binary-scored items, yielded a Cronbach’s alpha of 0.81 during pilot testing. Additionally, the health behavior scale, consisting of 10 Likert-type items, exhibited a test–retest intraclass correlation coefficient (ICC) of 0.79. An example of a knowledge item included: “What does ‘sodium’ on food labels indicate?” with response options: A. Energy, B. Protein, C. Salt content, and D. Fat. Inclusion criteria for participants required (1) parental or guardian consent, and (2) access to a smartphone to facilitate app-based interventions. No exclusion criteria were applied.

Prior to proceeding with registration or completing the questionnaire, participants were presented with an informed consent form was presented on the screen via the “Healthy Salt Classroom” applet. This form comprehensively outlined the study’s objectives, procedures, potential risks and benefits, the voluntary nature of participation, and the confidentiality measures implemented to ensure confidentiality. Participants, including the parents or legal guardians of minors, were required to thoroughly review the consent form before gaining access to the registration process or questionnaire. The consent procedure was systematically documented electronically, with timestamps and user identification details securely recorded in the database. This approach guaranteed that informed consent was obtained from all participants or their authorize representatives before their engagement in the study.

Between October 1, 2022, and May 31, 2023, participants took part in a dual-setting intervention comprising home-based and school-supported components. The home-based intervention included 20 animated lessons, each lasting 5 min (totaling 100 min), accompanied by quizzes delivered through a smartphone application. The school-supported intervention involved teacher-led activities, consisting of monthly 45-min sessions focused on salt reduction, including meetings and competitions. Of the original cohort, 11,614 students, representing a retention rate of 74.2%, completed both the baseline and post-intervention assessments.

### 2.2 Data collection

The selection of specific items for the health knowledge and behavior questionnaires was informed by a comprehensive preparatory phase to ensure cultural relevance, scientific rigor, and appropriateness for the target age group. This process included a review of local health education syllabi, focus group discussions with primary school health teachers from Zhenjiang, and, crucially, iterative consultations with a multidisciplinary panel of public health experts. This panel included specialists from the George Institute for Global Health at Peking University and the China Health Education Center (providing national strategic and methodological guidance), and George Institute for Global Health Research (contributing international perspectives, methodological guidance and theoretical rigor), Jiangsu Provincial Center for Disease Control and Prevention (providing regional practical insights). The variables (e.g., Four Pillars of Health, Handwashing Behavior) were prioritized through a consensus-based approach, grounded in their identified importance for the health literacy of Chinese primary school students and their precise alignment with the key intervention messages of the ‘Healthy Salt Classroom’ program. This collaborative method ensures that the measured constructs are contextually meaningful, scientifically sound, and directly relevant to the intervention’s objectives.

Prior to and following the course, students completed online questionnaires independently or with parental assistance through the “Healthy Salt Classroom” application. The questionnaires consisted of two components: health knowledge and health behavior, containing 20 and 10 multiple-choice items, respectively. Responses to both sections were scored and aggregated. In the health knowledge section, each correct response was assigned a value of 1 point, while incorrect responses received 0 points. For the health behavior section, each item was rated on a scale from 0 to 1, reflecting the extent of positive or negative behavior exhibited. Ultimately, scores for both health knowledge and behavior were expressed as percentages.

### 2.3 Intervention measures

The intervention comprised a comprehensive health education program delivered via a mobile application, targeting both students and their parents. The primary components were as follows: (1) the installation of the “Healthy Salt Classroom” applet on parents’ mobile devices, facilitating the joint completion of 20 health education lessons by students and parents. These lessons utilized animated narratives illustrating everyday scenarios at school and home, addressing topics such as balanced nutrition, personal hygiene, prevention of infectious diseases, and strategies for salt reduction. (2) The organization of diverse health education activities within schools to cultivate a supportive environment. (3) Provision of online training for School canteen chefs focused on salt reduction practices. (4) Implementation of salt reduction-themed meetings and extracurricular activities led by class teachers, with student participation incentivized through the accumulation of points in their accounts.

### 2.4 Quality control

Operational protocols were established, and comprehensive training sessions were conducted for project staff, school principals, and classroom teachers prior to the commencement of the intervention. Throughout the implementation phase, the “Healthy Salt Classroom” management application and website enabled real-time monitoring of course completion and activity documentation, thereby ensuring rigorous quality control.

### 2.5 Statistical analysis

Statistical analyses were performed utilizing R version 4.4.1 and IBM SPSS Statistics 25. Students’ participation scores were stratified into three categories according to course completion thresholds of 80 and 60%. This categorization follows common practice in educational and public health research, where a score of ≥80% is considered ‘good’ engagement, 60–79% represents ‘medium’ engagement, and <60% indicates ‘poor’ engagement. To assess differences between pre- and post-intervention scores, paired chi-square tests and paired t-tests were employed. Additionally, a mixed-effects model was implemented to examine the factors associated with changes in scores.

## 3 Results

A total of 15,656 students from 88 schools in Zhenjiang participated in the intervention, of whom 11,614 completed both the pre- and post-intervention surveys, resulting in a response rate of 74.2%. Among these participants, 90.1% exhibited good or average levels of engagement. Table 1 provides a comparative analysis of online course performance between students from urban and rural areas, revealing highlighting significant disparities in educational outcomes. Specifically, students residing in urban areas outperformed their rural counterparts, as indicated by a higher proportion of students attaining a ‘good’ performance status in urban settings (66.2% in cities versus 43.1% in counties).

**Table 1:** Post-intervention online course performance by area and district [*n* (%)].

Area	Number of schools	Number of students	According to academic performance by course* [*n*(%)]		
			Good	Medium	Poor
Live in					
Urban	37	6,592	4,361(66.2)	1737(26.4)	494(7.5)
Rural	49	5,022	2,165(43.1)	2,200(43.8)	657(13.1)
District					
Danyang	19	2003	587(29.3)	1,068(53.3)	348(17.4)
Jurong	21	3,193	2038(63.8)	975(30.5)	180(5.6)
Yangzhong	13	1746	1,298(74.3)	390(22.3)	58(3.3)
Jingkou	12	1810	1,054(58.2)	596(32.9)	160(8.8)
Runzhou	7	488	152(31.1)	235(48.2)	101(20.7)
Dantu	7	635	341(53.7)	211(33.2)	83(13.1)
New District	7	1739	1,052(60.7)	462(26.6)	221(12.7)
Total	86	11,614	6,526(56.2)	3,937(33.9)	1,151(9.9)

*Academic Performanc categorized by Course Grade Performance (Good: ≥80%; Medium: 60–79%; Poor: <60%).

The districts of Yangzhong, Jurong, and Jingkou demonstrated significantly elevated proportions of students attaining ‘good’ performance levels in online courses. In particular, 74.3% of students in Yangzhong, 63.8%in Jurong, and 58.2% in Jingkou were classified as performing well.

These statistics differ from the general average ‘good’ performance rate of 56.2% across all regions, suggesting that these specific districts have demonstrated notably greater effectiveness in their health education initiatives.

### 3.1 Changes in health knowledge and behavior

Subsequent to the health education intervention, there was a statistically significant increase in the awareness rates across all categories of health knowledge categories among the students, as presented in Table 2. Specifically, knowledge domains exhibiting an increase of exceeding 10% included Vision Protection Knowledge, underscoring the intervention’s efficacy in this area; the Correct Method of Coughing and Sneezing, reflecting enhanced understanding of respiratory health; and Recommended Salt Intake for Adults. It is noteworthy that, despite overall improvement in health knowledge awareness, substantial variability persisted across different topics. Baseline awareness levels for Optimal Bedtime for Primary Students, Benefits of Ventilation, and knowledge of Emergency Numbers were already above 93%, indicating a well-established foundational knowledge prior to the intervention. Conversely, the awareness rates for the Four Pillars of Health, Milk Intake for Children, and Recommended Salt Intake for Adults remained below 50% even after the intervention, highlighting the need for sustained educational efforts to promote comprehensive health literacy among students.

**Table 2:** Variations in health knowledge awareness among primary school students pre- and post- intervention [*n* (%)].

Health knowledge	Pre-intervention	Post-intervention	*χ*^2^ Value	*P*-value
Four pillars of health	1,776 (15.3)	2,068 (17.8)	521.2	<0.001
Optimal bedtime for primary students	10,932 (93.3)	11,171 (96.2)	682.8	<0.001
Dental care knowledge	8,897 (76.6)	9,991 (86.0)	428.2	<0.001
Vision protection knowledge	5,201 (44.8)	7,044 (60.7)	1380.0	<0.001
Knowledge of myopia	10,256 (88.3)	10,921 (94.0)	409.4	<0.001
Mental health knowledge	10,279 (88.5)	10,296 (88.7)	104.6	<0.001
Substances causing food poisoning	7,559 (65.1)	7,935 (68.3)	810.7	<0.001
Correct way to cough/sneeze	7,788 (67.1)	9,440 (81.3)	831.8	<0.001
Benefits of ventilation	11,312 (97.4)	11,352 (97.7)	62.8	<0.001
Lung function	9,766 (84.1)	10,167 (87.5)	1263.5	<0.001
Emergency numbers	11,331 (97.6)	11,320 (97.5)	409.8	<0.001
Knowledge of appropriate exercise	10,597 (91.2)	11,035 (95.0)	392.2	<0.001
Milk intake for children	2,786 (24.0)	3,252 (28.0)	265.9	<0.001
Recommended salt intake for adults	3,964 (34.1)	5,437 (46.8)	230.7	<0.001
Main component of salt	10,486 (90.3)	10,865 (93.6)	412.8	<0.001
Salt content in food	10,505 (90.5)	10,712 (92.2)	636.6	<0.001
Statements about salt	10,223 (88.0)	11,011 (94.8)	116.5	<0.001
Statements about salt reduction	10,193 (87.8)	10,882 (93.7)	408.4	<0.001
Recognition of sodium in food labels	10,286 (88.6)	10,843 (93.4)	1208.8	<0.001
Calculation of sodium in nutrition labels	7,276 (62.6)	8,152 (70.2)	1023.5	<0.001

Table 3 illustrates the variations in health behavior scores among primary school students prior to and following the intervention. While the majority of the health behavior habits demonstrated improvement post-intervention, no statistically significant differences were observed in Oral Hygiene Behavior and Breakfast Consumption Behavior. A detailed analysis further indicated that the increases in health knowledge scores were more pronounced than those in health behavior scores, suggesting that the acquisition of knowledge may subsequent behavioral modifications.

**Table 3:** Variations in health behavior scores among primary school students pre- and post-intervention (Mean ± standard deviation, points).

Health behavior	Pre-intervention	Post-intervention	*t*-value	*P*-value
Handwashing behavior after school	94.5 ± 15.2	95.2 ± 14.0	4.8	<0.001
Vision check behavior	92.5 ± 20.5	94.8 ± 16.9	10.8	<0.001
Oral hygiene behavior	85.63 ± 22.6	85.90 ± 22.5	1.0	0.313
Daily sleep time	74.7 ± 27.1	79.9 ± 26.4	17.9	<0.001
Behavior of choosing packaged food	90.3 ± 29.5	94.6 ± 22.5	14.0	<0.001
Breakfast consumption behavior	98.1 ± 11.8	98.2 ± 11.5	0.5	0.602
Dietary taste preferences	64.2 ± 28.0	70.5 ± 28.0	20.8	<0.001
Consumption of salty snacks	83.2 ± 27.9	86.5 ± 24.7	11.0	<0.001
Exercise behavior	77.2 ± 28.1	79.6 ± 27.5	8.1	<0.001
Eating out or ordering takeaway	82.3 ± 18.9	83.2 ± 18.3	4.8	<0.001

Following the intervention, the mean scores for students’ health knowledge and health behavior increased by 5.6 and 2.6 points respectively, resulting in an overall average increase of 4.4 points in combined health knowledge and behavior scores. Improvements were observed regardless of whether parents assisted students in responding to questions before and after the intervention, the scores improved after the intervention. Notably, students who independently completed the assessments both prior to and following the intervention demonstrated the most substantial gain in health knowledge scores, with an increase of 12.4 points, Conversely, students who received parental assistance at baseline but completed the final assessment independently exhibited the greatest improvement in health behavior scores, with an increase of 2.8 points, as detailed in Table 4.

**Table 4:** Scores of health knowledge and behavior pre- and post- intervention (x ± s, points).

Survey condition	Number of respondents	Pre-intervention	Post-intervention	*t*-value	*P*-value
Overall health knowledge score	11,614	73.8 ± 13.0	79.2 ± 12.0	46.0	<0.001
Independently answered by students	796	66.5 ± 17.6	78.9 ± 15.6	31.5	<0.001
Baseline student answered, final parents assisted	884	67.4 ± 16.6	75.5 ± 14.1	15.4	<0.001
Baseline parents assisted, final student answered *	1,798	72.7 ± 13.6	76.2 ± 13.6	9.8	<0.001
Parents assisted both times *	8,136	75.4 ± 11.3	80.8 ± 10.4	42.8	<0.001
Overall health behavior score	11,614	84.3 ± 11.6	86.9 ± 10.9	23.9	<0.001
Independently Answered by Students	5,103	85.1 ± 11.9	87.6 ± 11.4	15.1	<0.001
Baseline student answered, final parents assisted	1,561	83.8 ± 12.1	86.5 ± 10.4	8.9	<0.001
Baseline parents assisted, final student answered	2,611	84.1 ± 10.7	86.9 ± 10.6	12.1	<0.001
Parents assisted both times	2,339	83.0 ± 11.3	85.4 ± 10.6	11.0	<0.001
Overall health knowledge and behavior score	11,614	77.3 ± 10.4	81.7 ± 10.0	48.4	<0.001

Variations between pre-intervention and post-intervention scores were evaluated using ANOVA (F = 124.8, *P* < 0.001; F = 12.5, *P* < 0.001). Subsequent multiple comparisons were performed employing Dunnett’s test, designating independent student responses as the control condition. An asterisk (*) denotes a statistically significant difference relative to the control group (*p* < 0.005).

### 3.2 Determinants affecting score variations

**Figure 1:**
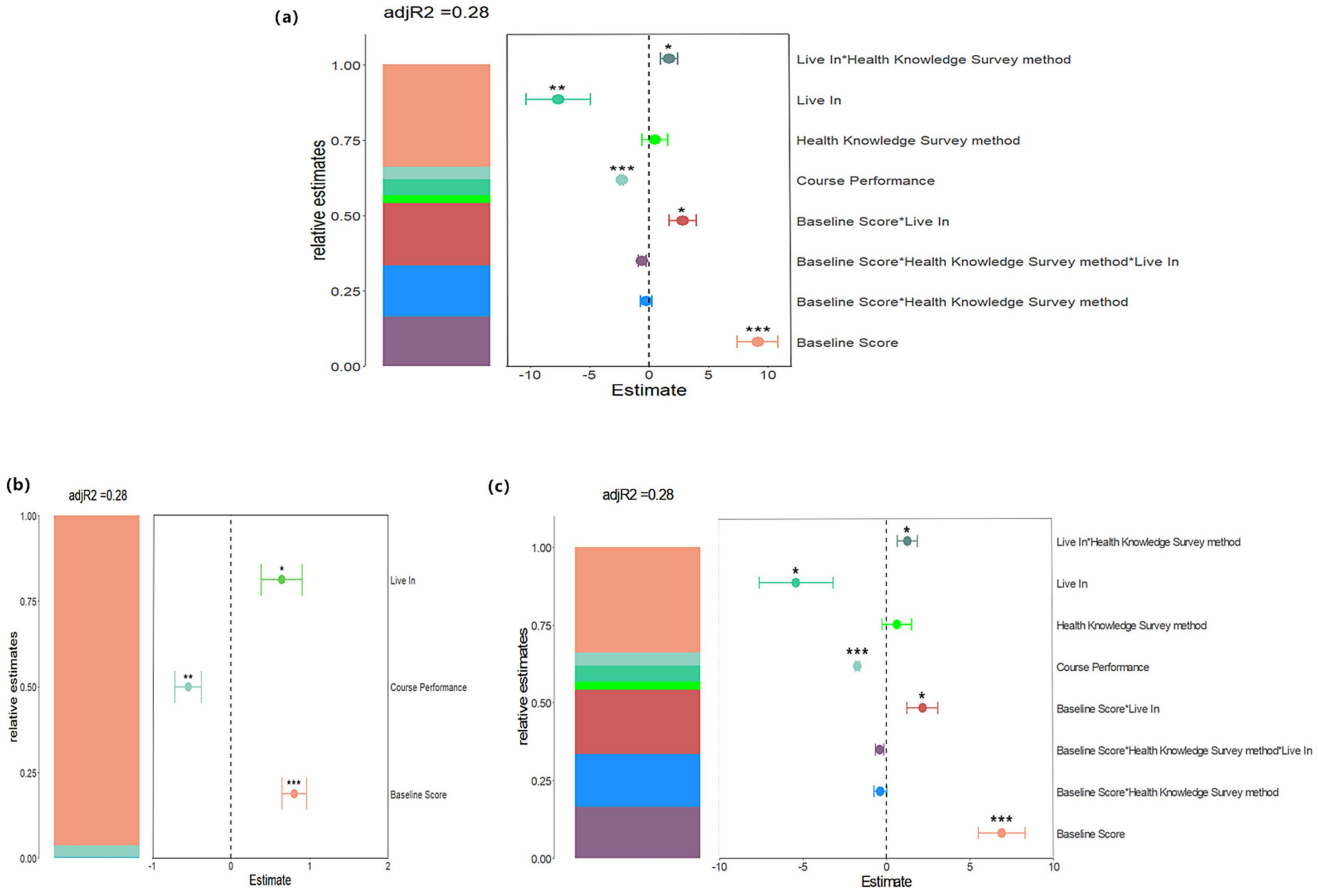
Mixed Effects Model Analysis of the Influence of Various Variables on Relative Estimates. Panels **(a–c)** depict the effects of changes in health knowledge, behavior, and their combined influence on relative estimates, respectively, with an adjusted R^2^ value of 0.28. In each panel, the left-side bar presents the relative estimates for different variables, while the scatter plot on the right displays the relationship between these estimates and the corresponding variables. Asterisks denote levels of statistical significance.

Figure 1 and Table 5 illustrate the results derived from a mixed effects model examining alterations in health knowledge and behavior scores subsequent to intervention. This analytical approach incorporates both fixed effects—such as the intervention’s direct impact— random effects, which account for including individual differences, group-level variability, and their interactions. The baseline score exerts a significant influence on health knowledge score (*β* = 9.12, 95% CI: 5.74 to 12.49, *p* < 0.001), health behavior score (*β* = 0.80, 95% CI: 0.50 to1.11, *p* < 0.001), and the total scores (*β* = 6.90, 95% CI: 4.15 to9.65, p < 0.001). This finding suggests that students with higher initial scores tend to exhibit smaller improvements following the. Academic performance demonstrates a negative association with health knowledge score (*β* = −2.29, 95% CI: −2.60 to −1.98, p < 0.001), health behavior score (*β* = −0.54, 95% CI: −0.87 to −0.21, *p* = 0.001), and total score (*β* = −1.74, 95% CI: −2.00 to-1.48, p < 0.001). Similarly, residing in rural areas is negatively correlated with health knowledge (*β* = −7.64, 95% CI: −12.93 to −2.38, *p* = 0.005), health behavior (*β* = −0.65, 95% CI: 0.14 to 1.16, *p* = 0.019), and total scores (*β* = −5.41, 95% CI: −9.75 to −1.11, *p* = 0.014). These results indicate that students with lower academic achievement those living in rural settings experience less pronounced score improvements post-intervention. Furthermore, the interaction between the baseline score and the area of residence significantly affects health knowledge score (*β* = 2.85, 95% CI: 0.62 to5.09, *p* = 0.012) and the total score (*β* = 2.15, 95% CI: 0.33 to 3.97, *p* = 0.021), suggesting that the influence of the baseline scores on these outcomes may vary depending on students’ residential context. Additionally, the interaction between the area of residence and the method used to assess health knowledge significantly impacts the health behavior scores (*β* = 1.69, 95% CI: 0.25 to 3.13, *p* = 0.036), indicating that the effect of the survey method on health behavior outcomes may be contingent upon the students’ area of residence.

**Table 5:** Mixed effects model analyzing for factor influencing changes in health knowledge and behavior scores following the intervention.

Variable	Outcome	*β* (95% CI)	*P*-value
Baseline score	Health knowledge change	9.12 (5.74, 12.49)	< 0.001 ***
	Health Behavior Change	0.80 (0.50, 1.11)	< 0.001 ***
	Total Score Change	6.90 (4.15, 9.65)	< 0.001 ***
Course performance	Health knowledge change	−2.29 (−2.60, −1.98)	< 0.001 ***
	Health Behavior Change	−0.54 (−0.87, −0.21)	0.001 **
	Total Score Change	−1.74 (−2.00, −1.48)	< 0.001 ***
Health knowledge survey method	Health knowledge change	0.48 (−1.65, 2.60)	0.661
	Total Score Change	0.62 (−0.15, 1.39)	0.484
Residence (live in)	Health knowledge change	−7.64 (−12.93, −2.38)	0.005 **
	Health Behavior Change	−0.65 (−1.16, −0.14)	0.019 *
	Total Score Change	−5.41 (−9.75, −1.11)	0.014 *
Baseline score × survey method	Health knowledge change	−0.23 (−1.17, 0.72)	0.638
	Total Score Change	−0.37 (−1.14, 0.39)	0.342
Baseline score × residence	Health knowledge change	2.85 (0.62, 5.09)	0.012 *
	Total Score Change	2.15 (0.33, 3.97)	0.021 *
Survey method × residence	Health knowledge change	1.69 (0.25, 3.13)	0.022 *
	Total Score Change	1.26 (0.08, 2.43)	0.036 *

*CI, confidence interval. Significance levels: *p < 0.05, **p < 0.01, **p < 0.001. The reference category for ‘Residence’ is (e.g., Urban). ‘Course Performance’ is defined as (e.g., a continuous grade point average).

## 4 Discussion

This study has several important limitations, with the absence of a concurrent control group representing the most significant constraint on causal inference from our pre-post comparisons. Although we observed substantial improvements in knowledge and behaviors, we cannot exclude the possibility that these changes resulted from external factors—including natural maturation, concurrent health campaigns, or test–retest effects—rather than our intervention alone. This design limitation was unavoidable given the city-wide administrative implementation, where withholding the program from eligible students was not ethically feasible. To address this constraint partially, we employed mixed-effects models controlling for baseline scores and key demographic variables.

Despite these limitations, our findings offer encouraging preliminary evidence that comprehensive health education programs enhanced with mobile health technologies may substantially improve students’ health knowledge and behavioral outcomes. Future controlled studies are essential to establish definitive causal relationships and confirm these promising initial results.

The present study demonstrates that a health education intervention conducted over two academic semesters significantly improved both health knowledge and behaviors among primary school students. These findings corroborate prior research indicating that traditional pedagogical approaches, as well as mobile application-based health education strategies, can effectively enhance health-related behaviors in students and their families (22, 23). In this investigation, we utilized a novel series of animated instructional modules, successfully integrating them into the school curriculum, thereby establishing the feasibility and efficacy of mobile health-based comprehensive interventions in student health education. The data reveal an approximate 4% increase in the overall health knowledge scores of primary school participants. A systematic review of mobile health interventions targeting children and adolescents reported an average behavioral improvement effect size of 0.22, with an average intervention duration of 4.8 months (24, 25). The comparatively smaller effect size observed in this study may be attributable to the broad scope of knowledge assessed and the extended intervention period of 12 months, which could have resulted in partial knowledge attrition by the time of the final evaluation. Furthermore, the increment in health knowledge scores slightly exceeded that of health behavior improvements, suggesting that behavioral modification may present greater challenges than knowledge acquisition (26, 27). Given that salt reduction constituted a primary focus of the curriculum, students demonstrated superior mastery and assessment performance in salt-related knowledge relative to other health education topics. Additionally, a positive correlation was observed between students’ engagement levels in the program and the magnitude of improvement in both health knowledge and behaviors, indicating that active participation is a critical determinant of educational outcomes (28).

Considering that the intervention was aimed at third and fourth grade students, who are relatively young, the intervention model was developed to engage both students and their parents via a mobile mini-program, thereby inherently incorporating parental support in responding to questions. To elucidate the influence of parental involvement and to differentiate between independently completed student responses and completed with parental assistance, the research team classified response patterns into distinct categories. The findings demonstrated score improvements irrespective of parental assistance, suggesting that participation in the program was advantageous for both students and their parents.

This project addressed salt reduction, a significant public health concern, as its focal point, and encompassed the essential knowledge outlined in health education guidelines for primary and secondary schools. By integrating both online and offline participation from schools and families, the initiative offered innovative approach to enhancing health education contemporary educational settings (29). The adoption of a flexible and efficient mobile health education strategy facilitated convenient for monitoring and evaluation processes and was recognized as an effective tool for promoting behavioral change (30). Nonetheless, certain left-behind children residing with their grandparents encountered challenges in accessing online courses due to limited smartphone availability, thereby necessitating continued reliance on school-based health education. Furthermore, students’ acquisition of health knowledge and behaviors has the potential to positively influence their families, encouraging healthy dietary practices, weight management, obesity prevention, and the reduction of cardiovascular disease risk. Future applications of this model could be extended to other health domains where awareness remains insufficient (31).

Utilizing a mixed effects model analysis, this study investigated the dynamics of health education interventions in improving the health knowledge and behaviors of primary school students. The findings indicated several important outcomes: students with higher baseline scores demonstrated a smaller increase in health knowledge and behaviors following the intervention, implying that the marginal benefit of the intervention may be diminished for those who already possess a certain level of health literacy (32). Consequently, customizing interventions to align with students’ initial health literacy levels—by adapting content to b eboth challenging and supportive for learners at varying stages of health awareness, may enhance the overall efficacy of such programs (33, 34). Furthermore, the significant interaction observed between the area of residence and baseline health knowledge scores, as well as the methods used to assess health knowledge, suggests that the influence of these factors on health knowledge and behavior may be contingent upon the residential context. This finding implies that environmental variables, such as urban versus rural settings, may affect effectiveness of health education initiatives.

Our findings can be effectively interpreted using the very behavioral frameworks that informed the intervention’s design. This phenomenon can be elucidated through behavioral change frameworks such as the intention-behavior gap (35) and the COM-B model, which conceptualizes behavior (B) as the product of the interplay among Capability, Opportunity, and Motivation (35). The intention-behavior gap helps explain why forming an intention to change is fundamentally easier than implementing it into sustained action. In the present intervention, the primary focus was on augmenting students’ capability (C) by imparting knowledge via animated instructional materials. Nonetheless, the translation of increased capability into enduring behavioral modification may have been impeded by constraints related to opportunity (O)—for instance, limited availability of healthy food choices at home or the incomplete implementation of salt reduction policies in school cafeterias—and motivation (M), including entrenched taste preferences for salty foods and social influences from peers (36). Consequently, future interventions should extend beyond purely educational strategies to incorporate more robust, theory-driven behavioral techniques. Such approaches might encompass environmental restructuring aimed at facilitating healthier choices (e.g., ensuring the accessibility of low-sodium food options) and the application of persuasive technologies designed to bolster motivation through mechanisms such as goal-setting, self-monitoring, and feedback provision (37). Moreover, as underscored in a recent review by Liang et al. (2025), advancements in food science—particularly the utilization of food colloids—present promising avenues for reducing sodium content in processed foods without detriment to taste perception (38). The integration of these technological innovations with educational efforts holds potential to mitigate critical environmental barriers and more effectively reconcile the gap between knowledge acquisition and behavioral enactment in forthcoming public health interventions.

Beyond nutritional science and broader environmental restructuring, the digital interface itself plays a pivotal role. Furthermore, user experience (UX) represents a critical determinant of engagement efficacy and intervention success in mHealth platforms. The observed disparities in engagement patterns and health outcomes between urban and rural student populations may be partially attributable to differential UX interactions with the ‘Healthy Salt Classroom’ applet. Critical UX parameters—including navigational intuitiveness, visual design aesthetics, animation interactivity, and compatibility with legacy smartphone hardware prevalent in rural households—constitute significant mediators of sustained user motivation and platform utilization. Enhanced UX design directly augments motivational capacity (the ‘M’ component within the COM-B framework), thereby facilitating the critical knowledge-to-behavior translation pathway. Future mHealth intervention development would benefit substantially from systematic integration of evidence-based UX design principles and rigorous usability validation protocols, particularly through comprehensive testing across diverse socioeconomic user cohorts, to ensure equitable engagement and optimize intervention effectiveness across heterogeneous populations (39).

The findings offer valuable insights into the domain of school health education and bear significant implications for both policy formulation and practical implementation. Future investigations should seek to overcome the limitations identified in this research by incorporating extended follow-up durations, designing control, and broadening the scope to include diverse geographic regions and demographic populations. Such approaches will facilitate a more nuanced understanding of the determinants influencing the efficacy of health education initiatives, thereby providing an empirical foundation for the development of optimized health education strategies.

## Data Availability

The datasets presented in this article are not readily available because confidentiality: the dataset contains information about individual students and their families, so confidentiality must be maintained. Identifiable personal information cannot be disclosed or shared publicly. Ethical approval: the study was approved by ethics committees, which imposed conditions on how the data could be collected, stored, and used. Researchers must adhere to these ethical guidelines when working with the dataset. Informed Consent: Participants and their parents provided informed consent specifically for this study. The dataset can only be used in ways that were agreed upon in the consent forms. Research Use Only: The dataset is restricted to research purposes directly related to the study’s objectives. It cannot be used for commercial purposes, marketing, or any other activities outside the scope of the approved research. Requests to access the datasets should be directed to lindsay731@sina.com.
